# HX-1171 attenuates pancreatic β-cell apoptosis and hyperglycemia-mediated oxidative stress via Nrf2 activation in streptozotocin-induced diabetic model

**DOI:** 10.18632/oncotarget.24916

**Published:** 2018-03-23

**Authors:** Jimin Kim, Su-Hyun Shin, Jong-Koo Kang, Jae Wha Kim

**Affiliations:** ^1^ Cell Factory Research Center, Division of Systems Biology and Bioengineering, Korea Research Institute of Bioscience and Biotechnology, Daejeon, Republic of Korea; ^2^ Department of Functional Genomics, KRIBB School of Bioscience, University of Science and Technology, Daejeon, Republic of Korea; ^3^ Department of Laboratory Animal Medicine, College of Veterinary Medicine, Chungbuk National University, Cheongju, Republic of Korea

**Keywords:** HX-1171, Nrf2 activator, beta cell, diabetes, hyperglycemia

## Abstract

Streptozotocin (STZ) acts specifically on pancreatic beta cells, inducing cell destruction and cell dysfunction, resulting in diabetes. Many studies have reported that nuclear factor-erythroid 2-related factor 2 (Nrf2), a main regulator of antioxidant expression, prevents and improves diabetes-related diseases. In this study, we investigated the antidiabetic effect of the newly discovered Nrf2 activator, HX-1171, in the STZ-induced diabetic mouse model. HX-1171 enhanced insulin secretion by reducing STZ-induced cell apoptosis, and decreased intracellular reactive oxygen species (ROS) generation by upregulating the expression of antioxidant enzymes through Nrf2 activation in INS-1 pancreatic beta cells. In STZ-induced diabetic mice, HX-1171 administration significantly lowered blood glucose levels and restored blood insulin levels. In the STZ-only injected mice, the pancreatic islets showed morphological changes and loss of function, whereas the HX-1171-treated group was similar to that of the control group. These results suggest that HX-1171 may be developed as a promising therapeutic agent for diabetes-related diseases.

## INTRODUCTION

Streptozotocin (STZ) has a glucose-like structure and enters the pancreatic beta cells through GLUT2, a glucose transporter, causing alkylation and fragmentation of DNA, generation of reactive oxygen species, activation of caspase-3-PKCδ-IL-1β apoptotic signal pathways, and fatal damage to the cells [1, 2]. The dysfunction of pancreatic beta cells induced by these actions of STZ results in a diabetic condition. Diabetes is a metabolic disease that can cause many serious abnormalities including hyperglycemia. Chronic hyperglycemia leads to three major complications [3, 4]: retinopathy, nephropathy, and neuropathy. That many diabetic patients suffer from these complications shows the risk of persistent hyperglycemia [5, 6].

Failure to regulate blood glucose levels causes hyperglycemia-mediated oxidative stress, which induces apoptosis in pancreatic beta cells [7]. Studies have reported that the expression of antioxidant enzymes is lower in the pancreas than in other tissues [8, 9]. In fact, oxidative stress specifically in the pancreas has been shown in the diabetic mouse model [10, 11] and diabetic patients [12, 13]. The intake of natural foods with antioxidant effects, such as herbs and vegetables, has been recognized as beneficial to diabetics [14]. The action of antioxidant enzymes against oxidative stress in the pancreas also has been closely associated with slowing the progression of diabetes and reducing the occurrence of diabetic complications [15].

The major regulator mediating the expression of antioxidants is transcription factor nuclear factor-erythroid 2-related factor 2 (Nrf2). Nrf2 is activated by oxidative stress, translocating to the nucleus and upregulating the expression of its target genes. A number of studies have reported that Nrf2 plays an important role in the prevention and treatment of diabetes [16–19]. Moreover, Nrf2 has been demonstrated as effective in the treatment of diabetes-related complications such as retinopathy [20–22], nephropathy [23–25], and neuropathy [26, 27]. In addition, genetic Nrf2 induction significantly reduced beta cell damage in the reactive oxygen species (ROS)-mediated mouse model of diabetes, whereas genetic Nrf2 deficiency resulting from conditional-Nrf2 knockout markedly aggravated beta cell damage [28]. Therefore, we investigated the antidiabetic effect of HX-1171, a newly discovered Nrf2 activator [29], in the INS-1 pancreatic beta cell line and the STZ-induced diabetic mouse model.

HX-1171 is a non-cytotoxic compound that enhances the expression of antioxidant enzymes very effectively by promoting nuclear translocation of Nrf2 [29]. In the pancreatic beta cell line, INS-1, HX-1171 diminished rate of cell apoptosis and generation of intracellular ROS induced by STZ treatment. In the STZ-induced diabetic mouse model, the elevated blood glucose levels were reduced in the HX-1171-treated group, and HX-1171 protected pancreatic beta cells from acute tissue damage. These results suggested that HX-1171 attenuated apoptosis and hyperglycemia-mediated oxidative stress in pancreatic beta cells through Nrf2 activation. Therefore, HX-1171 may have a therapeutic and preventive effect on diabetes-related diseases.

## RESULTS

### The STZ-induced reduction in pancreatic beta cell viability was reversed by HX-1171

**Figure 1:**
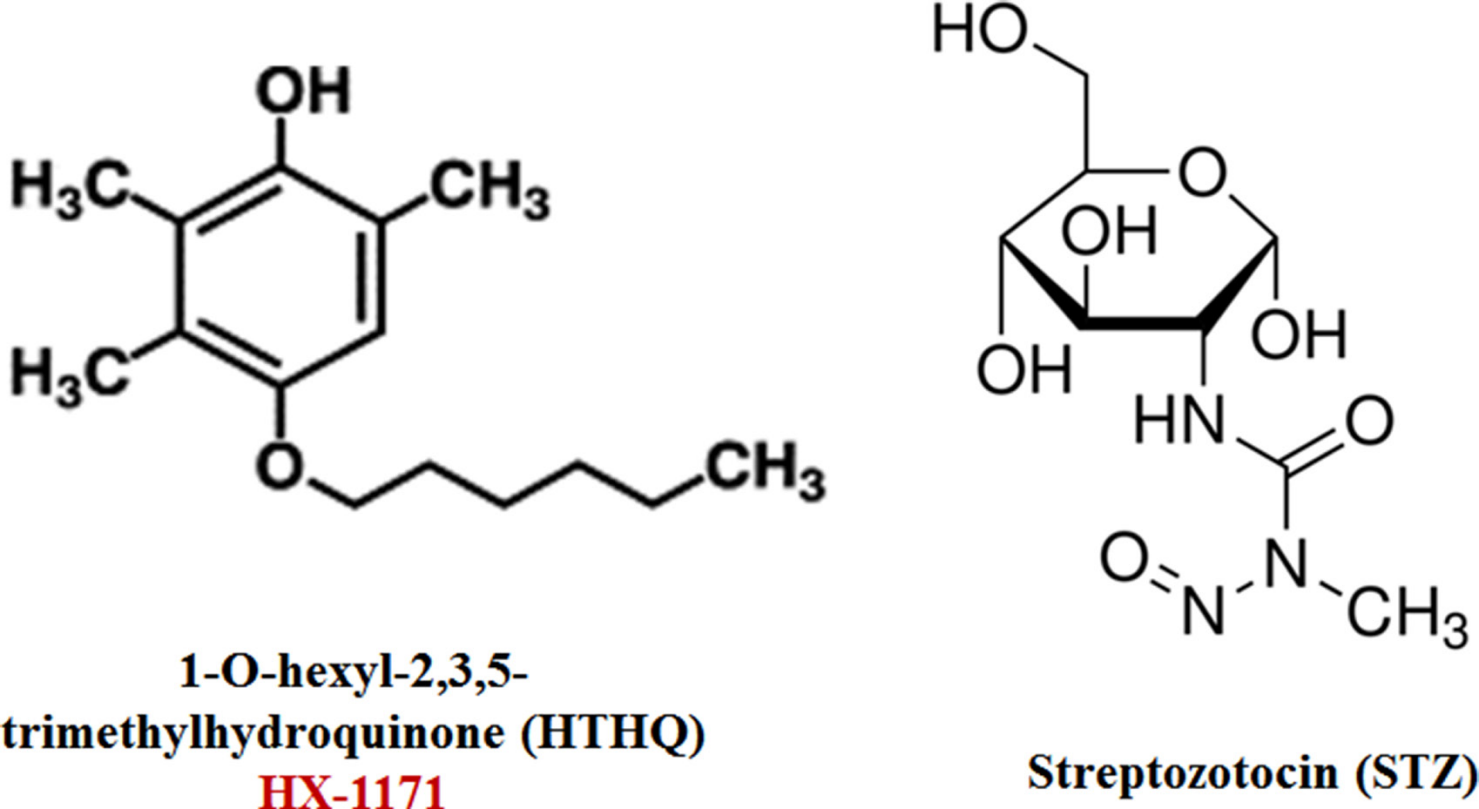
Chemical structures of HX-1171 and Streptozotocin

**Figure 2:**
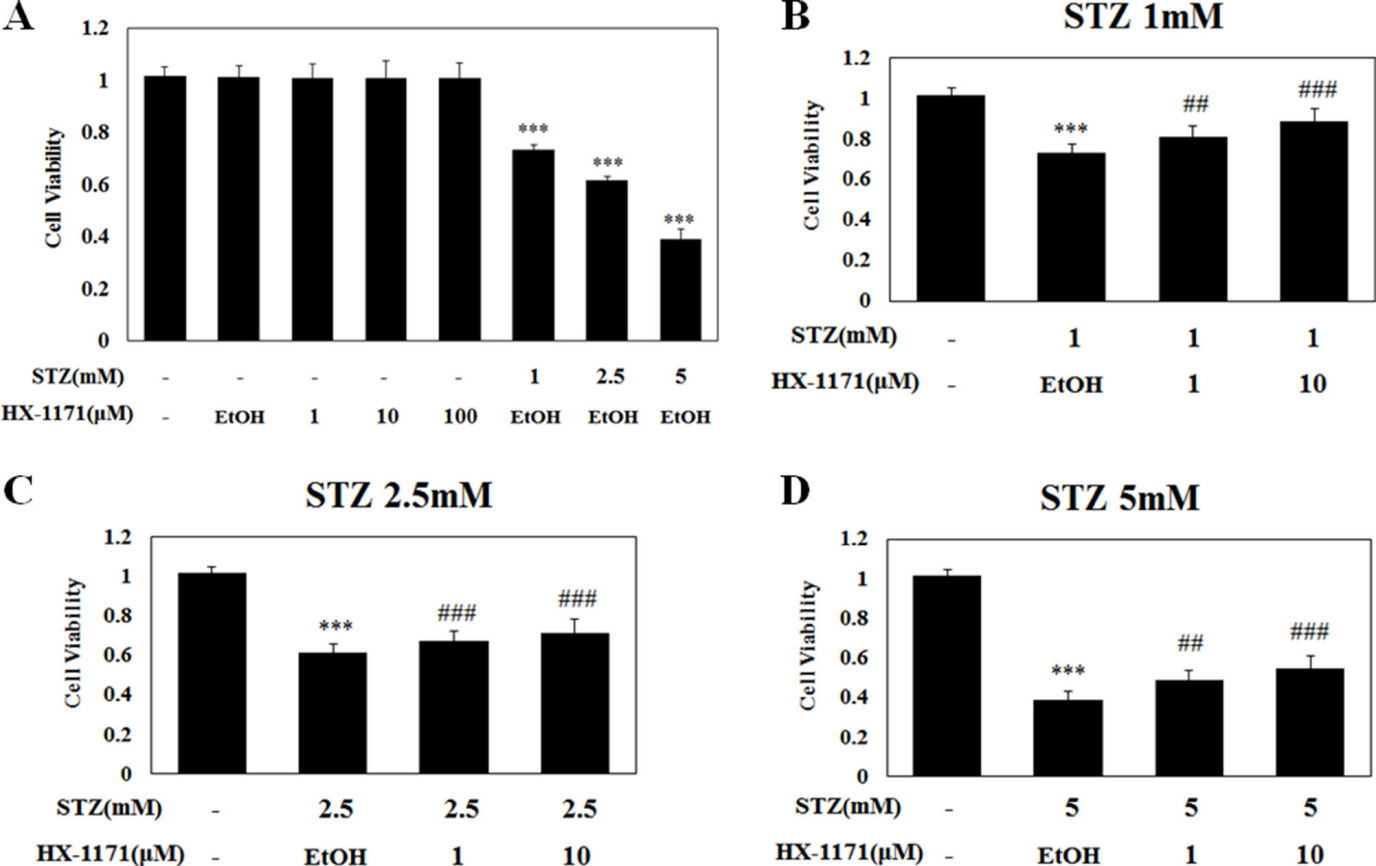
Effect of HX-1171 on cell viability of STZ-treated cells (**A**) INS-1 cells were either untreated or treated with (**B**) 1 mM, (**C**) 2.5 mM, and (**D**) 5 mM of STZ and indicated dose of HX-1171 for 24 hrs. Cell viability was analyzed by WST-1 assay. Data are presented as mean ± SD (*n* = 4). ^***^P < 0.001 compared to the control group, ^#^*P* < 0.05, ^###^*P* < 0.001 compared to the STZ group.

The chemical structures of HX-1171 and STZ are shown in Figure 1. Cell viability was analyzed by WST assay. INS-1 cells were treated with HX-1171 and STZ (Figure 2A). No cytotoxicity was observed when cells were treated with HX-1171 alone, but cell viability was decreased by STZ treatment. Treatment of cells with STZ at doses of 1 mM (Figure 2B), 2.5 mM (Figure 2C), and 5 mM (Figure 2D), reduced cell viability, which was reversed by co-treatment with HX-1171 in a dose-dependent manner.

### HX-1171 reduced STZ-induced cell apoptosis

**Figure 3:**
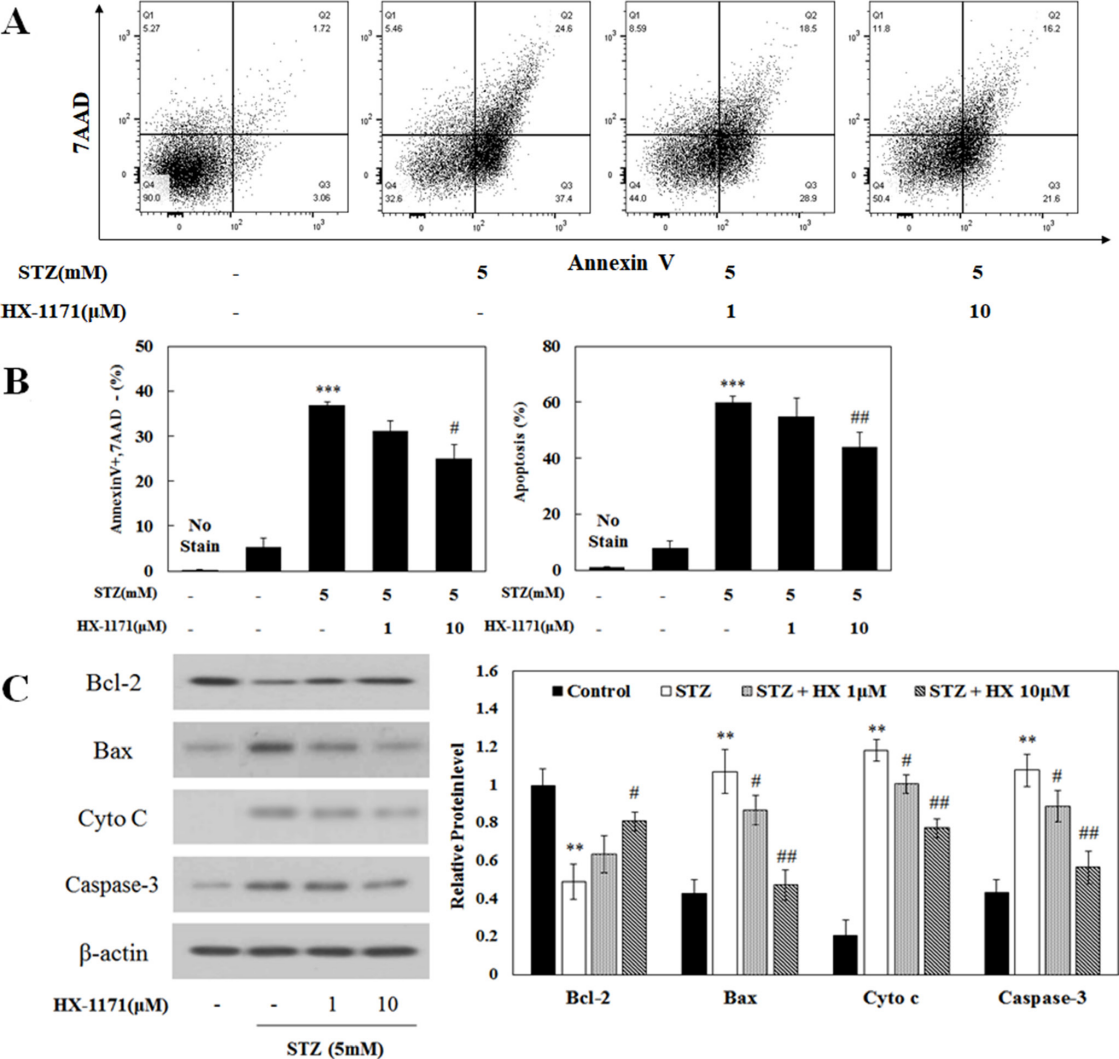
Effect of HX-1171 on STZ-induced cell apoptosis Cell apoptosis was analyzed by flow cytometry of cells double-stained with Annexin V and 7-AAD. (**A**) Flow cytometric dot plots, and (**B**) Annexin-V-positive/7-AAD-negative (early apoptotic) and total apoptosis of STZ and HX-1171-treated INS-1 cells. The cells were either untreated or treated with STZ (5 mM) and indicated dose of HX-1171 for 16 hrs. (**C**) Expression of apoptosis-related signal proteins was analyzed by western blotting. INS-1 cells were either untreated or treated with STZ (5 mM) and indicated doses of HX-1171 for 4 hrs. Protein levels were normalized to β-actin. Data are presented as mean ± SD (*n* = 3). ^**^P < 0.005, ^***^P < 0.001 compared to the control group, ^#^*P* < 0.05, ^##^*P* < 0.005 compared to the STZ group.

Cell apoptosis was analyzed by flow cytometry. Dot plots of cells positive for Annexin V and 7-AAD are shown in Figure 3A. The ratio of apoptotic cells was increased by STZ treatment and that effect was reduced by HX-1171 treatment (Figure 3B). In addition, expression of apoptosis-related proteins was analyzed by western blot (Figure 3C). Bcl-2, an anti-apoptotic protein, was decreased by STZ and increased by HX-1171 treatment. Expression of apoptosis-related proteins such as Bax, cytochrome c, and caspase-3 was increased by STZ and decreased by HX-1171 treatment. Relative levels of proteins were normalized to β-actin and quantification was performed using ImageJ software.

### HX-1171 reduced STZ-induced intracellular ROS generation

**Figure 4:**
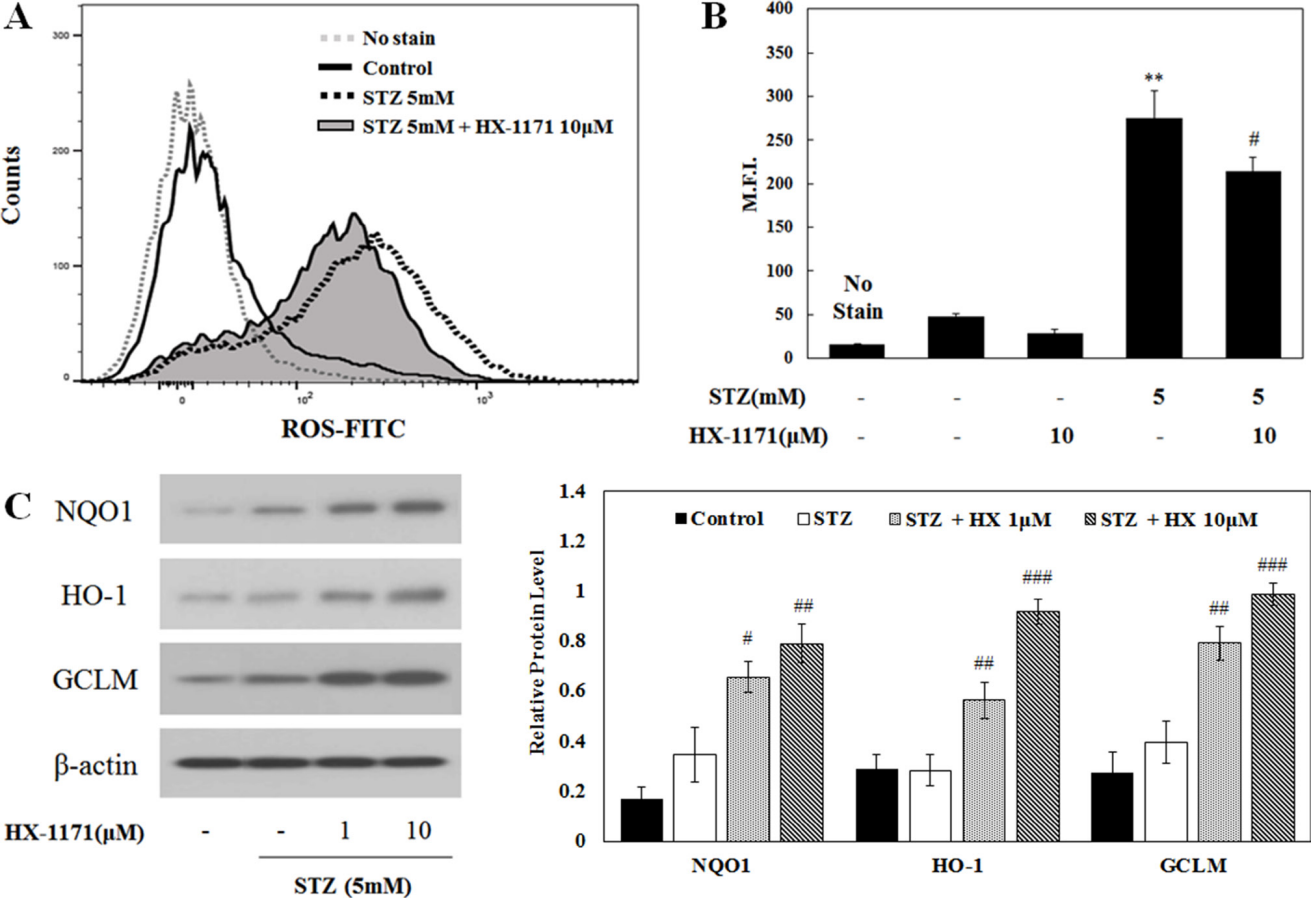
Effect of HX-1171 on STZ-induced intracellular ROS expression (**A**) Intracellular ROS levels and (**B**) Mean Fluorescence Intensity (M.F.I.) were analyzed by flow cytometry using DCFH-DA dye. Cells were either untreated or treated with STZ (5 mM) and HX-1171 (10 μM) for 16 hrs. (**C**) Expression of Nrf2-related antioxidant proteins was analyzed by western blotting. INS-1 cells were either untreated or treated with STZ (5 mM) and indicated doses of HX-1171 for 4 hrs. Protein levels were normalized to β-actin. Data are presented as mean ± SD (*n* = 3). ^**^P < 0.005 compared to the control group, ^#^*P* < 0.05, ^##^*P* < 0.005, ^###^*P* < 0.001 compared to the STZ group.

Intracellular ROS expression was analyzed by flow cytometry (Figure 4A). Histograms showing the increase of intracellular ROS expression showed a shift in the mean fluorescence intensity (M.F.I.) to the right in cells treated with STZ, but the degree of shift was decreased by HX-1171 treatment. In addition, the M.F.I. (Figure 4B) in STZ-treated cells was reduced by HX-1171 treatment. To demonstrate the effect of HX-1171 on intracellular ROS expression, the expression of antioxidant enzymes, which are targets of Nrf2, was analyzed by western blot (Figure 4C). The expression of NAD(P)H quinone oxidoreductase 1 (NQO1), heme oxygenase-1 (HO-1), and γ-glutamate cysteine ligase modifier subunit (GCLM) was increased by HX-1171 treatment. Relative levels of protein expression were normalized to β-actin and quantification was performed using ImageJ software.

### HX-1171 improved insulin secretion in INS-1 cells

**Figure 5:**
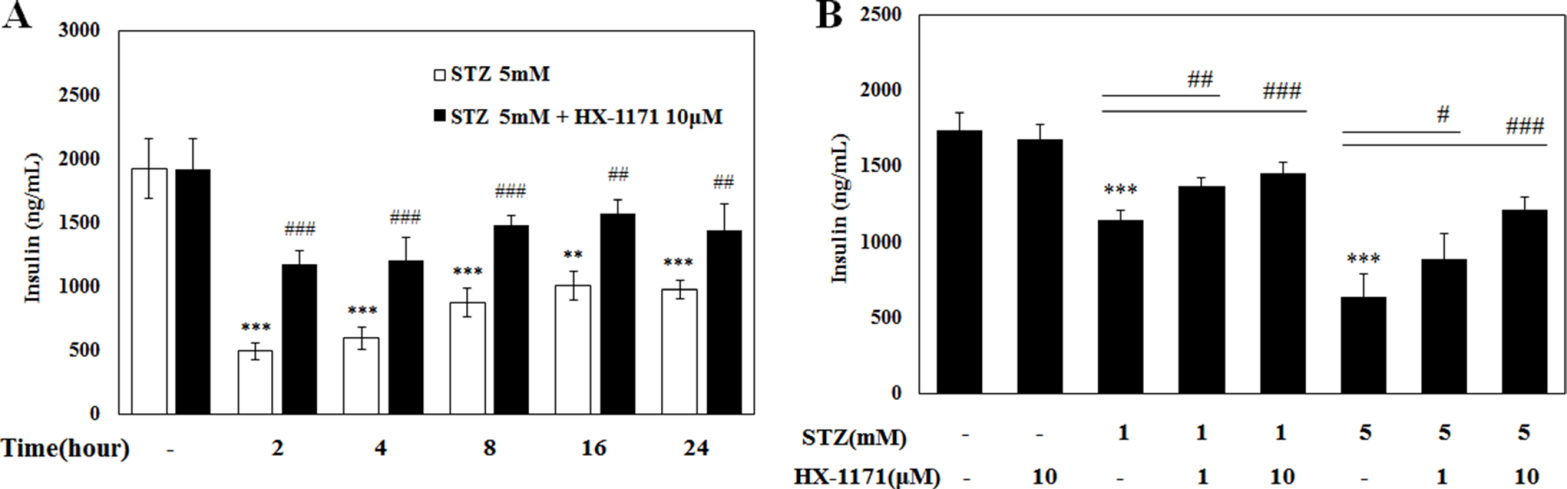
Effect of HX-1171 on insulin secretion by STZ-treated cells Insulin secretion was measured by ELISA. (**A**) INS-1 cells were either untreated or treated with STZ (5 mM) and HX-1171 (10 μM) for the indicated times, and (**B**) the cells were treated with the indicated dose of STZ and HX-1171 for 2 hrs. Data are presented as mean ± SD (*n* = 4). ^**^P < 0.005, ^***^P < 0.001 compared to the control group, ^#^*P* < 0.05, ^##^P < 0.005, ^###^*P* < 0.001 compared to the STZ group.

After incubation of INS-1 cells with STZ and HX-1171 at the indicated times, culture media were collected and the insulin secretion was analyzed by ELISA (Figure 5A). Over time, levels of secreted insulin accumulated and increased. The amount of insulin in the HX-1171-treated cells was always higher than that in the STZ-only treated cells. Subsequently, cells were treated STZ and HX-1171 for 2 h and insulin secretion was increased by HX-1171 in a dose-dependent manner (Figure 5B).

### HX-1171 protected pancreatic beta cells from STZ-induced damage

**Figure 6:**
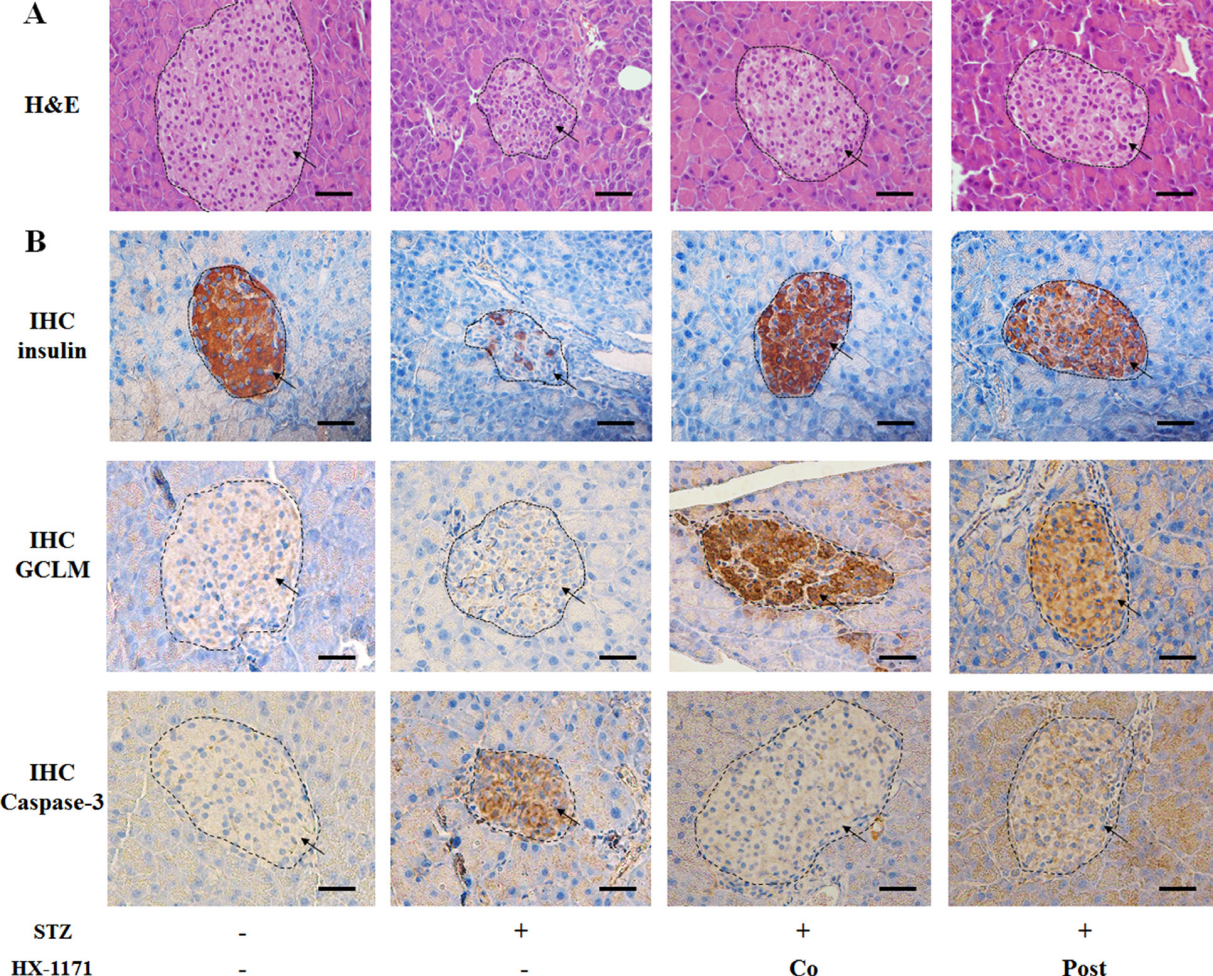
Effect of HX-1171 on pancreatic beta cells in the STZ-induced diabetic mouse model (**A**) Hematoxylin and eosin (H&E)-stained pancreas sections in untreated, STZ-only treated (200 mg/kg, i.p.), HX-1171-co-treated (125 mg/kg, p.o.), and HX-1171-post-treated (p.o. one day after STZ injection) mice. (**B**) Insulin-, GCLM-, and caspase-3-expressing cells in the pancreas were analyzed by immunohistochemistry. All images (Figure 6A and 6B) are shown at 400× magnification. Scale bar = 50 μm.

Pancreas tissues were stained with H&E and evaluated histologically. The STZ-treated pancreatic beta cells showed damage and were shrunken compared to the controls (Figure 6A). In the HX-1171 treatment groups, both the co-treated group and the post-treated group showed less damage than the STZ-only treated group. When the expression of insulin, GCLM, and caspase-3 in the pancreatic beta cell was examined by immunohistochemistry (Figure 6B), the numbers of insulin- and GCLM-positive cells in HX-1171-treated groups were higher, and caspase-3-positive cells were lower than the STZ group.

### HX-1171 lowered high blood glucose levels

**Figure 7:**
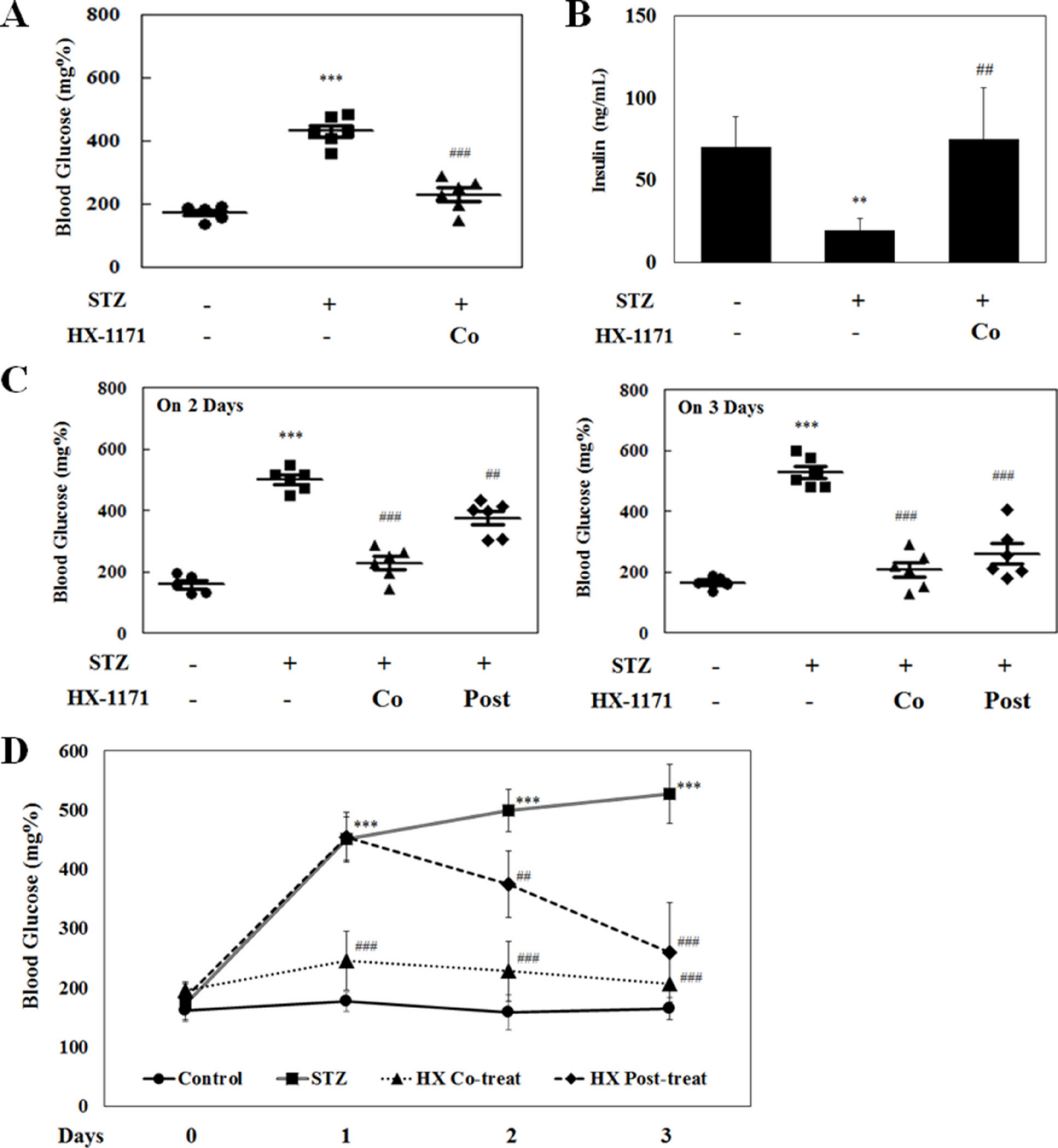
Effect of HX-1171 on blood glucose levels in the STZ-induced diabetic mouse model (**A**) Blood glucose levels and (**B**) serum insulin levels in untreated, STZ (200 mg/kg, i.p.) and HX-1171 (125 mg/kg, p.o.)-co-treated mice 1 day after STZ treatment. (**C**) Blood glucose levels in untreated, STZ, HX-1171-co-treated and HX-1171-post-treated (p.o. one day after STZ injection) mice at the indicated days. (**D**) The average level of blood glucose in mice at the indicated days. Blood glucose levels were measured using an Accu-Chek glucometer, and serum insulin levels were measured by ELISA. ●: Control (*n* = 5), ■: STZ (*n* = 6), ▲: HX-1171-co-treat (*n* = 6), ♦: HX-1171-post-treat (*n* = 6). Data are presented as mean ± SD. ^**^P < 0.005, ^***^P < 0.001 compared to the control group, ^##^*P* < 0.005, ^###^*P* < 0.001 compared to the STZ group.

In the STZ-induced diabetic mouse model, blood glucose was measured on the first day after STZ injection (Figure 7A). The blood glucose levels were remarkably (*P* < 0.001) increased in STZ-only injected group, but not in the HX-1171-treated groups. Blood insulin levels were measured by ELISA (Figure 7B). The secreted insulin level was decreased in the STZ-treated group, whereas the insulin level in HX-1171-treated group was similar to that of the control group. In addition, post-treatment with HX-1171 significantly reduced the blood glucose levels that were increased by STZ injection (Figure 7C, 7D).

### HX-1171 improved blood glucose uptake during oral glucose tolerance test (OGTT)

**Figure 8:**
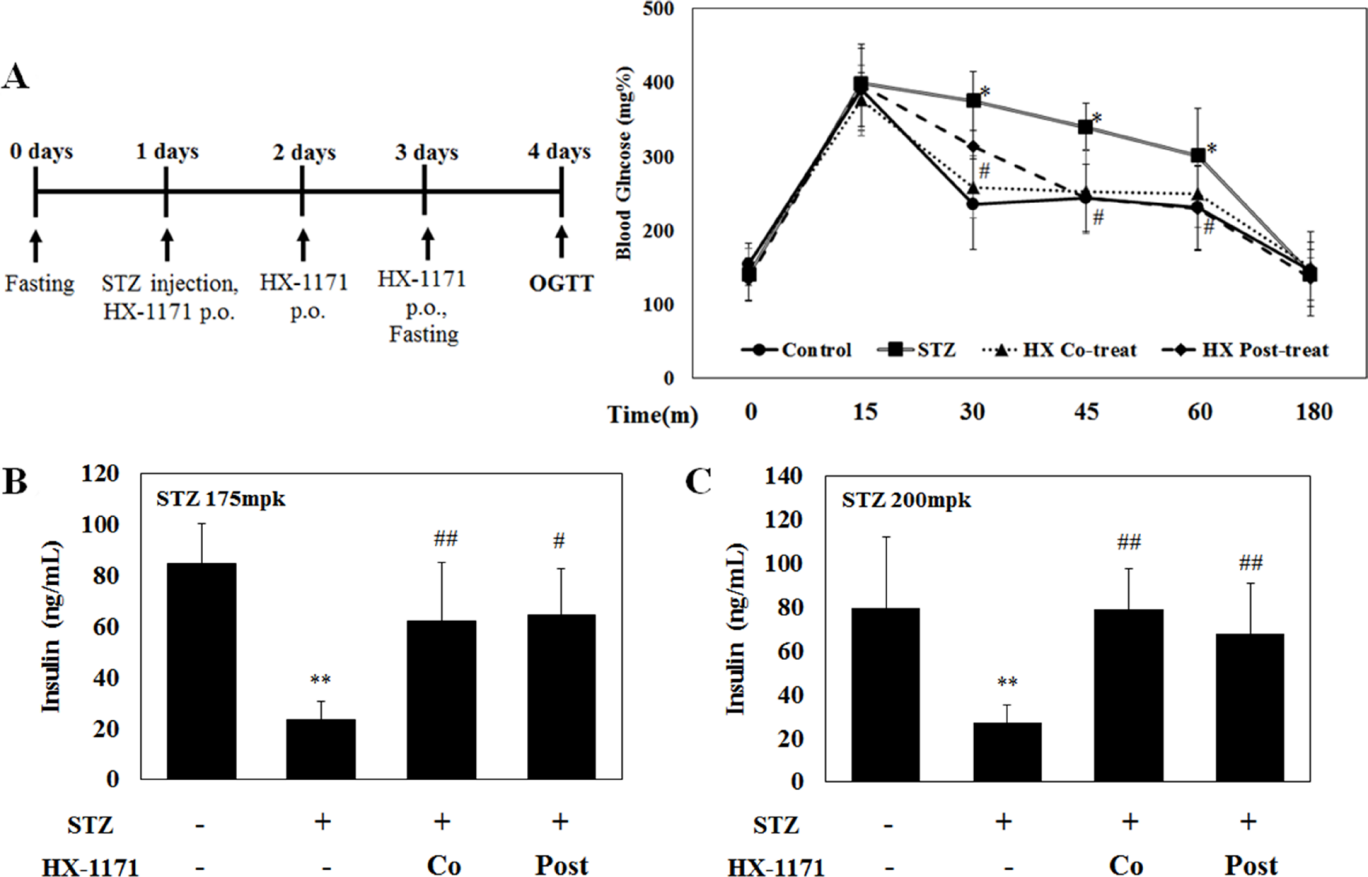
Effect of HX-1171 in STZ-induced diabetic mice during oral glucose tolerance test (OGTT) (**A**) The average level of blood glucose in mice either untreated or treated with STZ (175 mg/kg or 200 mg/kg, i.p.), HX-1171-co-treated (125 mg/kg, p.o.), and HX-1171-post-treated (p.o. one day after STZ injection) during the OGTT were determined to ascertain the ability of mice to process a bolus dose of glucose. Blood glucose levels were measured using the Accu-Chek Diabetes Monitoring Kit. Serum insulin levels in mice (at 30 min, during OGTT) injected with STZ (**B**) 175 mg/kg and (**C**) 200 mg/kg were analyzed by ELISA. ●: Control (*n* = 5), ■: STZ (*n* = 6), ▲: HX-1171-co-treat (*n* = 6), ♦: HX-1171-post-treat (*n* = 6). Data are presented as mean ± SD. ^*^P < 0.05, ^**^P < 0.005 compared to the control group, ^#^*P* < 0.05, ^##^*P* < 0.005 compared to the STZ group.

OGTT was performed in the control, STZ, HX-1171 co-treated, and HX-1171 post-treated groups to ascertain blood glucose uptake. Before the test, the mice were fasted for 20 h, and then were given 2000 mg/kg of D-glucose p.o. Blood glucose levels were measured at the indicated times (Figure 8A). At 15 min after glucose administration, the highest blood glucose level was observed in all groups, which began to decrease beginning at 30 min. The STZ group showed the lowest blood glucose reduction and the HX-1171 group showed significantly enhanced glucose reduction in the co-treated and post-treated groups compared to the control group. For induction of diabetes in the mice, STZ concentrations used were 175 mg/kg (Figure 8B) and 200 mg/kg (Figure 8C). STZ injection decreased the level of insulin in the blood, while the insulin levels were higher in the co-treated and post-treated groups.

## DISCUSSION

The cytotoxic action of STZ on pancreatic beta cells has been studied extensively. The pancreas is more susceptible to STZ because it is particularly sensitive to glucose and most highly expresses GLUT2, which acts as a channel for glucose and STZ [30, 31]. GLUT2 is also weakly expressed in liver and kidney, so these organs could be damaged by STZ. The nitrosourea moiety of STZ is a substantial cause of its cytotoxic activity, acting as a donor of intracellular NO [32, 33]. Furthermore, STZ activates expression of inducible nitric oxide synthase (iNOS), and c-jun N-terminal kinase (JNK)/p38 mitogen-activated protein kinase (MAPK) signaling pathways [34]. STZ is stable under acidic conditions and degrades DNA strands through interaction with cytosine residues of DNA in the pH 5–5.5 range [35]. DNA fragmentation leads to inflammation, and infiltration of inflammatory immune cells into the pancreas is one of the causes of progressive destruction of pancreatic beta cells [36, 37]. Furthermore, when beta cells are destroyed, insulin secretion is reduced, resulting in hyperglycemia. Excessive glucose metabolism due to elevated blood glucose levels promotes the glucose oxidation and intracellular ROS production [38, 39]. Excessive production of intracellular ROS induces oxidative stress, and persistent hyperglycemia and hyperglycemia-mediated oxidative stress can lead to diabetes and fatal complications.

Currently, Nrf2 activators are attracting attention due to their role in preventing and alleviating diabetes-related diseases. Nrf2, a transcription factor mediating gene expression of antioxidant enzymes, is important for cellular defense mechanisms against hyperglycemia-mediated oxidative stress and cellular damage [40]. In a previous study, we discovered a new Nrf2 activator, HX-1171. HX-1171 showed superiority in increasing the expression of Nrf2 target genes compared to tBHQ, another Nrf2 activator, and a remarkable effect in promoting nuclear translocation of Nrf2 [29]. Thus, we investigated the efficacy of HX-1171 in an STZ-induced diabetic model to confirm whether HX-1171 could be effective for diabetic conditions like other Nrf2 activators.

In our *in vitro* results, we found that HX-1171 protected INS cells against STZ-induced apoptosis, and diminished STZ-induced intracellular ROS generation by increasing the expression of antioxidant enzymes through Nrf2 activation. Western blotting results revealed that HX-1171 suppressed the expression of apoptosis-related proteins and increased the expression of antioxidant enzymes. In addition, HX-1171 improved the secretion of insulin by STZ-damaged beta cells in a dose-dependent manner. In the *in vivo* mouse model, HX-1171 administration significantly ameliorated STZ-induced pancreas beta cell damage, and effectively reduced the elevated blood glucose levels. Furthermore, HX-1171 administration restored the blood insulin levels after STZ injection, and improved the blood glucose uptake ability of diabetic mice during OGTT.

The number of patients suffering from diabetes and its complications is steadily increasing [41, 42], especially in industrialized countries, where the number of diabetic patients have been estimated at 10% of the total population. The incidence of diabetes continues to increase due to an extension in human longevity, a westernized diet, and lack of exercise [43, 44]. Many therapies developed so far have not provided effective treatments, and as the diabetes population grows, new and more effective therapies need to be developed. In this respect, the effect of HX-1171 on lowering the high blood glucose levels and improving insulin secretion in STZ-induced diabetic model is very promising, and our results suggest that HX-1171 may be applied and developed as a therapeutic agent for diabetes-related diseases.

## MATERIALS AND METHODS

### Cell culture

Rat insulin-secreting pancreatic beta cells (INS-1) were obtained from Dr. Jung, Korea Research Institute of Bioscience and Biotechnology (KRIBB, Daejeon, Korea). Cells were cultured in RPMI-1640 medium (Welgene, Gyeongsangbuk-do, Korea) containing 10% fetal bovine serum (FBS), 50 μM β-mercaptoethanol, 100 units/mL penicillin, and 100 μg/mL streptomycin. The cells were grown in a humidified atmosphere in 5% CO_2_ at 37°C.

### Chemicals and reagents

HX-1171 (1-O-hexyl-2,3,5-trimethylhydroquinone) was obtained from Biotoxtech Co., Ltd. (Cheongju, Korea), and streptozotocin (STZ) was purchased from Enzo Life Sciences (Farmingdale, NY, USA). HX-1171 was dissolved in ethanol, and STZ was dissolved in culture medium then used for *in vitro* treatment. The final working concentration of ethanol was 0.1% (v/v). For *in vivo* experiments, HX-1171 was dissolved in a solvent mixture of 8% (v/v) Tween 80, 5% (w/v) NaCl, and distilled water; STZ was dissolved in 0.1 M citrate buffer (pH 4.5).

### Cell viability assay

Cell viability was measured by water-soluble tetrazolium salt-1 (WST-1) assay. Briefly, INS-1 cells were seeded into 96-well plates and treated with STZ (1 mM, 2.5 mM and 5 mM) and HX-1171 (0.1 mM, 1 mM and 10 mM) for 24 hrs. Cell viability was measured by adding 10 mL of D-Plus™ CCK reagent (Dongin LS, Seoul, Korea). The cells were incubated for 1 h at 37°C, and the optical density (OD) at 450 nm was determined using an EMax Precision Microplate Reader (Molecular Devices, Sunnyvale, CA, USA).

### Western blotting

INS-1 cells were seeded into 6-well plates and treated with STZ (5 mM) and HX-1171 (1 mM and 10 mM) for 4 hrs. The cells were lysed in RIPA buffer (LPS Solution, Daejeon, Korea) supplemented with protease and phosphatase inhibitors (Thermo Scientific, Waltham, MA, USA). Proteins were separated through a 12% sodium dodecyl sulfate-polyacrylamide gel and transferred to a polyvinylidene difluoride membrane (*Immobilon*-P Millipore, Darmstadt, Germany). The membranes were blocked with 5% bovine serum albumin (BSA) for 1 h, and incubated with primary antibodies (dilution 1:1000) to Bax (BS1030), Bcl-2 (BS1031) (Bioworld Tech, St. Louis Park, MN, USA), Cytochrome c (#4272), Caspase-3 (#9662) (Cell Signaling Technology, Danvers, MA, USA), NQO1 (sc-16464), GCLM (sc-22754), HMOX1 (sc-10789), and b-actin (sc-1616) (Santa Cruz Biotechnology, Dallas, TX, USA). After three washes with phosphate buffered saline with Tween 20 (PBST), membranes were incubated with horseradish peroxidase (HRP)-conjugated second antibodies (Santa Cruz Biotechnology, dilution 1:5000) for 1 h at room temperature. Protein bands were detected using ECL reagent (Thermo Scientific) and then visualized on film. ImageJ software was used for quantification of protein expression levels.

### Enzyme-linked immunosorbent assay (ELISA)

Anti-insulin antibody (dilution 1:200, ab8304, Abcam, MA, USA) in PBS was used to coat 96-well microtiter plates at 4°C overnight. Plates were washed three times with PBST and the wells were blocked with 2% BSA at room temperature for 1 h, followed by addition of samples. After incubation for 2 h, plates were washed three times with PBST, and HRP-conjugated anti-insulin antibody (dilution 1:1000, ab28063, Abcam) was added. Following a 1-hr incubation and three washes, 100 μL of tetramethylbenzidine (TMB) substrate solution was added to each well. The reaction was terminated by adding 100 μL 2 M sulfuric acid and the absorbance at 450 nm was recorded on an EMax Precision Microplate Reader (Molecular Devices).

### Flow cytometry

INS-1 cells were seeded into 12-well plates, and treated with STZ (5 mM) and HX-1171 (1 mM and 10 mM) for 16 hrs. Cells were collected by trypsinization and washed with PBS. For cell apoptosis analysis, INS-1 cells were incubated with Annexin V (5 mL per test, BD Biosciences, Franklin Lakes, NJ, USA) for 10 min at room temperature, and stained with 7-AAD (5 mL per test, BD Biosciences). For intracellular ROS analysis, the cells were incubated with 2 mM of DCFH-DA (Invitrogen, Carlsbad, CA, USA) for 30 min at 37°C. The analysis was performed using a BD FACS Verse flow cytometer (BD Biosciences).

### Diabetic mouse model

Ten- to twelve-week-old male BALB/c mice from KOATECH (Gyeonggi-do, Korea) were used and divided into four groups (5 to 6 mice per group): control, STZ, HX-1171-co-treat and HX-1171-post-treat group. After a 16-hr fast, all three groups of mice, except the control group, were injected intraperitoneally with STZ (175 mg/kg or 200 mg/kg) prepared fresh in citrate buffer. STZ group mice received no additional treatment. Beginning on the same day, HX-1171-co-treat group mice were treated with HX-1171 (125 mg/kg, p.o.) once daily for 3 consecutive days. HX-1171-post-treat group mice were treated with HX-1171 for 2 consecutive days beginning 1 day after STZ injection. Blood was collected from mice via the retro-orbital plexus, and changes in blood glucose level were monitored during the experiment. Blood glucose was measured using the Accu-Chek Diabetes Monitoring Kit (Roche, Seoul, Korea). All mice were sacrificed on day 4 and tissues were collected and fixed in 10% formalin for further analysis. All animal experiments were approved by the Institutional Animal Care and Use Committee of the Korea Research Institute of Bioscience and Biotechnology, and were performed in accordance with the Guide for the Care and Use of Laboratory Animals.

### Histopathology of pancreas islets

Pancreas tissues were fixed in 10% formalin, embedded in paraffin, and sectioned at 4 μm thickness. Sections were placed on slide glasses, stained with hematoxylin and eosin, and then observed under light microscope (Olympus, Tokyo, Japan). For immunohistochemistry, sections were deparaffinized and dehydrated using xylene and graded ethanol series. Staining was performed using the REAL^™^ EnVision^™^ Detection System Peroxidase-DAB kit (Dako, CA, USA) following the manufacturer's instructions. Anti-insulin antibody (ab8304, dilution 1:200) was from Abcam, anti-GCLM antibody (sc-22754, dilution 1:100) was from Santa Cruz Biotechnology, and anti-caspase-3 antibody (#9662, dilution 1:1000) was from Cell Signaling Technology.

### Oral glucose tolerance test (OGTT)

Prior to oral glucose tolerance testing (OGTT), mice were fasted for 20 h with free access to water. D-glucose was administered to the mice at a dose of 2000 mg/kg, p.o. and blood glucose levels were measured at 0, 15, 30, 45, 60 and 180 min using an Accu-Chek glucometer (Roche). During the test, blood collected at the 30-min time used to measure serum insulin levels by ELISA.

### Statistical analysis

Data are presented as means ± standard deviations. Differences between means were analyzed by Student's *t*- test. A *P* value of < 0.05 was considered statistically significant.
